# Anterior Spinal Artery Syndrome in a Patient with Cervical Spondylosis Demonstrated by CT Angiography

**DOI:** 10.1111/os.12555

**Published:** 2019-11-03

**Authors:** Ting Peng, Zheng‐feng Zhang

**Affiliations:** ^1^ Department of Orthopaedics, Xinqiao Hospital Third Military Medical University Chongqing China

**Keywords:** Anterior spinal artery, Anterior spinal artery syndrome, Cervical spondylosis, CT angiography

## Abstract

A few published reports have described anterior spinal artery syndrome (ASAS) with cervical spondylosis based on clinical presentation and/or MRI study, but no photographs of anterior spinal arteries were provided in these studies. Here we present a case of ASAS with cervical spondylosis in a CT angiography (CTA) study. A previously healthy 31‐year‐old man was diagnosed with acute ASAS with cervical spondylosis. Neurological examination revealed four‐limb weakness predominant in the distal part of the upper limbs and superficial sensory impairment below the cervical region. T2‐weighted images on MRI showed an area of hyperintensity in the gray matter of the cervical cord from C_3_ to C_5_ with a disc herniation at the C_4,5_ vertebral level. CTA demonstrated that ASA was occluded at level C_4,5_, which coincided with the location of disc herniation. Anterior spinal cord decompression and fusions were performed. The patient tolerated the procedure well and had complete resolution of his exertionally dependent myelopathic symptoms 1 week later. In conclusion, although ASAS with cervical spondylosis is rare, it can be diagnosed based on clinical symptoms and MRI and identified by CTA of ASA. A good neurological prognosis is anticipated after anterior spinal cord decompression and fusion is performed if disc herniation is responsible for ASA occlusion.

## Introduction

Anterior spinal artery syndrome (ASAS) is an extremely rare cause of acute ischemic cord infarction, which usually presents as an acute and painful myelopathy. It is caused by occlusion or hypoperfusion of the anterior spinal artery (ASA), which supplies the anterior two‐thirds of the spinal cord. ASAS is most commonly associated with thoracic/abdominal aortic‐related pathology, arteriovenous malformation, vasculitis, and iatrogenic causes^1^. The ASA run along the entire length of the anterior surface of the spinal cord. Therefore, ASA may theoretically be compressed by a herniated disc, resulting in spinal cord ischemia or infarct, which present as the high signal intensity area of the spinal cord on T2‐weighted MRI and in the low signal intensity area on T1‐weighted images, so called ASAS.

However, few cases of cervical spondylosis^2^, ^3^, ^4^, ^5^, ^6^, ^7^, ^8^, ^9^ and thoracic disc herniation^10^, ^11^, ^12^ leading to ASAS have been reported. The diagnosis of these reported cases was mostly based on clinical presentation^2^, ^3^ and MRI study^4^, ^5^, ^6^, ^7^, ^8^, ^9^, ^10^, ^11^, ^12^. Among them, spinal angiography images were only used for diagnosis in two cases^7^, ^10^. In this study, we presented a case of ASAS with cervical spondylosis using CT angiography (CTA) identification of ASA.

## Case Presentation

### *History*

A 31‐year‐old man presented to the emergency department and then transferred to the orthopaedic department complaining of inability to move his legs for 24 h. The patient stated that he had felt slight numbness in his legs and hands for 2 years and then began to feel weak while getting up from bed 24 h prior to presenting at the emergency department. This weakness quickly progressed until he was unable to stand and he fell to ground. He also reported transient neck pain for approximately 10 min at a time. The patient was a professional driver. He had not experienced recent injury or illness, headache, hypertension, or fever. His medical history was unremarkable.

### *Clinical Manifestation*

The patient's neurological examination revealed bilateral paralysis of the extremities. In the upper extremities he had 3/5 strength in the biceps and brachioradialis muscles, 3/5 in the wrist extensors, 3/5 in the triceps, and 3/5 in the hand extensors and flexors. In the lower extremities, he had 3/5–4/5 strength in all muscles. He had loss of light touch and pin prick sensation at the level of C_5_. Reflexes were absent in the lower extremities, biceps, brachioradialis, and triceps. Vibratory and positional senses were preserved throughout. All cranial nerves were intact. He had no vertebral point tenderness. The remainder of the patient's examination was unremarkable. The neurological status was evaluated as ASIA C. Because the mechanism of his paralysis was unclear at this time, the patient received 30 mg/kg of methylprednisolone over 50 min, followed by an infusion of 5.4 mg·kg^−1^·h^−1^ given over the next 23 h.

### *Imaging Findings*

**Figure 1:**
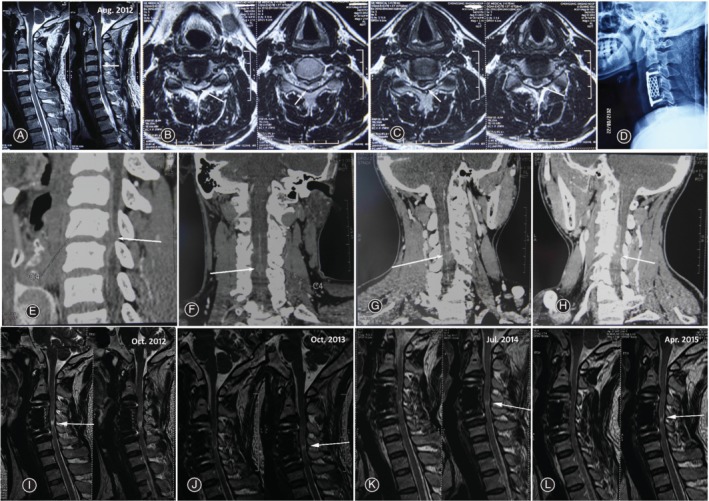
A 31‐year‐old man with ASAS and cervical spondylosis at C_3,4_ and C_4,5_. (A) Sagittal T2‐weighted MRI reveal that the cervical spinal cord was compressed at C_3,4_ and C_4,5_ (arrows). Spinal cord hyperintensity is showing from C_3_ to C_5_ (arrows). (B, C) Transverse T2‐weighted MRI showing spinal cord was compressed at C_3,4_ (B, arrows) and C_4,5_ (C, arrows). (D) Lateral radiograph view of anterior cervical corpectomy and fusion 3 days after surgery. (E, F, G, H) Sagittal view (E), coronal view (F), left oblique sagittal view (G), and right oblique sagittal view (H) of CT angiography showing anterior spinal artery occlusion at C_4,5_ (arrows). (I, J, K, L) The follow‐up MRI from 2 months (I), 14 months (J), 23 months (K) to 32 months (L) showing normal T2‐weighted imaging except for an area of myelomalacia cord at C_4,5_ (arrows).

Sagittal T2‐weighted MRI revealed that the patient presented spinal canal stenosis and the cervical spinal cord was compressed at the C_3,4_ and C_4,5_ (for almost 80%) intervertebral levels. Transverse T_2_‐weighted MRI demonstrated cord hyperintensity from C_3_ to C_5_ (Fig. 1A‐C). CTA presented ASA occlusion at C_4,5_ secondary to spinal disc herniation, which caused compression of the vascular supply (Fig. 1E‐H).

## Results

The patient was diagnosed with cervical compressive myelopathy combined ASAS, and undertook emergency anterior cervical corpectomy and fusion (Fig. 1D). The neurological grade improved dramatically from ASIA C to D 2 days later and to ASIA E 1 week later. The follow‐up MRI from 2 months to 3 years showed normal T2‐weighted imaging except for an area of cord myelomalacia at C_4,5_ (Fig. 1I‐L), the location of which was consistent with ASA occlusion.

## Discussion

Although ASA may theoretically be compressed due to a herniated disc and result in ASAS, only a few reports have described ASAS with cervical spondylosis and thoracic disc herniation^4^, ^5^, ^6^, ^7^, ^8^, ^9^, ^10^, ^11^, ^12^ (Table 1). However, most of these case reports were based on clinical presentation and MRI. Besides two cases using spinal angiography images^7^, ^10^ to my knowledge, this is only the third case of ASAS with cervical spondylosis diagnosed with ASA visual images.

**Table 1:** ASAS from cervical spondylosis and thoracic disc herniation based on clinical presentation, MRI images, and ASA angiography

Location	Author/year	Gender/Age (years)	Onset	Pain	Clinical presentation	Level	MRI	Intramedullary	Visualization of ASA	ASA occlusion	Diagnosis	Surgery	Prognosis
Cervical	Errea/1991^4^	M/49	Sudden	Lacinating In terscapular	Asymmetrical tetraparesis	C_6,7_	Disc protrusion	Plurisegmental anterior spinal lesion; C_6,7_	No	N/A	Clinical and MRI	Yes	Good
	Odaka/2004^5^	F/54	Acute	Angina	Progressive paraparesis	C_5–_C_7_	Disc herniation	Hyperintense; C_5_–C_7_	No VA abnormalities	N/A	Clinical and MRI	Yes	Good
	Shibuya/2005^6^	M/67;M/74;M/75	Acute	No	Arm paresis	C_3,4_; C_3–_C_7_; C_4–_C_6_	Cervical spondylosis	Intrinsic cord	No	N/A	Clinical, MRI and Electrophysiology	No	Good
	Arai/2007^7^	F/40	Acute	No	Bilateral four‐limb weakness	C_4,5_	Disc herniation	Hyperintensity; C_4,5_	Angiography	C4/C5	Clinical, MRI and ASA angiography	Yes	Good
	Ii/2009^8^	M/80	Sudden	Left shoulder pain	Hemiparesis to quadriparesis	C_3,4_	Disc herniation	Ischaemic lesion; C_2_–C_5_	No	N/A	Clinical and MRI	No	Poor
	Acker/2016^9^	F/72	Acute	No	Tetraparesis	C_5,6_	Disc herniation	Ischemia; C_3–_C_5_	No	N/A	Clinical and MRI	Yes	Good
Thoracic	Guest /2000^10^	F/38	Sudden	Back pain and radiculopathy	Paraparesis	T_8,9_	Disc herniation	Mildly increased signal; T_5,6_ to T_10,11_	Angiography	Strangulated	Clinical, MRI and ASA angiography	No	Good
	Yano/2003^11^	M/78	Sudden	No	Paraplegia	T_8,9_	Disc protrusion	High signal intensity; T_8_–T_9_	No	N/A	Clinical and MRI	No	Good
	Reynolds/2014^12^	F/36	Sudden	Back pain	Paraparesis	T_7,8_	Disc herniation	T2 hyperintensities and DWI brightness; T_4_–T_7_	No	N/A	Clinical, MRI and DWI	No	Good

ASA, anterior spinal artery; ASAS, anterior spinal artery syndrome; DWI, diffusion‐weighted magnetic resonance imaging; F, female; M, male; N/A, not applicable.

Diagnosis of ASAS is based on clinical examination, MRI findings, and visualization of ASA for confirmation. The typical presentation of ASAS is an acute and painful myelopathy. Clinical features include weakness and loss of pain and temperature sensation below the level of the involvement, with relative sparing of position and vibratory sensation. Bladder and bowel control are impaired in most cases^1^. The usual presentation of cervical spondylosis is a chronic compressive myelopathy, which is different from ASAS. The patient in the present study had both acute transient neck pain and a history of spinal cord compression at C_3,4_ and C_4,5_ (for almost 80% of the spinal canal sagittal diameter) and cord hyperintensity evident in MRI, which supports the diagnosis of cervical spondylosis combined with acute ASAS.

Cervical spondylosis, sometimes, may acquire over 80% spinal canal sagittal diameter compression and show T2‐weighted hyperintensity on MRI. It can be very difficult to identify the anterior cord if it is compressed and thinning. Therefore, the diagnosis of cervical spondylosis combined with ASAS was not only based on the patient's history and clinical symptoms, but, most importantly, on ASA occlusion. The patient in this study presented with over 80% spinal canal sagittal diameter compression and T2‐weighted hyperintensity on MRI, which was not a typical ASA infarct MRI because the anterior cord was compressed to the posterior. ASA occlusion in this patient was caused by compression of a massive disc herniation, which was removed 1 day after admission, with good neurological improvement.

Visualization of ASA can depict spinal cord blood supply in a normal spinal cord and to some extent spinal cord pathological ischemia. Catheter, CTm, and MR angiography are the ideal imaging techniques for diagnosing, localizing, and classifying spinal vascular lesions. CTA allows visualization of the cord and bone anatomy. That is the reason why we chose CT angiography for visualization of ASA, although its disadvantages include inherent exposure to ionizing radiation and administration of a potentially nephrotoxic iodine contrast agent^13^.

The exact cause of ASA occlusion for cervical spondylosis does not seem to be associated with the size of the herniated disc^4^, ^5^, ^6^, ^7^, ^8^, ^9^, ^10^, ^11^, ^12^ (Table 1). Because ASA infarct or occlusion is not commonly seen in cervical spondylosis patients with spinal canal sagittal diameter compression of less than 80%^13^ and acute blunt cervical spinal cord injury^14^, this indicates that the spinal cord is more easily compression compared with ASA in its anterior surface. Although we believe that ASA occlusion in this patient was caused by compression of a massive disc herniation, the other reported cervical spondylosis and thoracic disc herniation cases presented with small herniated discs on MRI^4^, ^5^, ^6^, ^7^, ^8^, ^9^, ^10^, ^11^, ^12^ (Table 1).

The treatment of ASAS is directed at the underlying cause, if known. Surgeries had been performed for some, but not all, reported severe cervical spondylosis and thoracic disc herniation cases^4^, ^5^, ^6^, ^7^, ^8^, ^9^, ^10^, ^11^, ^12^ (Table 1). In the present case, anterior cervical corpectomy and fusion was performed to remove the herniated disc, which was responsible for the compression of the ASA and the spinal cord. Generally, the response to high dose corticosteroids seems to be efficient; however, there are some patients who show a poor response^1^. The timing of the treatment is the most important determinant of the prognosis; namely, early invention leads to better outcomes. In the present case, both high dose steroid and anterior cervical corpectomy fusion surgery were administered early in the course of treatment, with resultant improvement in the neurological symptoms.

The long‐term prognosis of ASAS from many causes is poorly described^1^. However, neurological recovery occurs in most reported cervical spondylosis and thoracic disc herniations, especially for decompression surgery cases^4^, ^5^, ^6^, ^7^, ^8^, ^9^, ^10^, ^11^, ^12^ (Table 1). The present case improved dramatically from ASIA C to D 2 days after surgery and to ASIA E 1 week later. ASA reperfusion and decompression may be the reason for the improvement and good prognosis of neurological status for the cervical spondylosis combined ASAS patient.

### *Conclusion*

Anterior spinal artery syndrome with cervical spondylosis is rare and presents with an acute, painful myelopathy. Visualization of ASA by CTA can be identify ASA occlusion in ASAS with cervical spondylosis. The good neurological prognosis for the patient in the current study was encouraging following anterior spinal cord decompression and fusion performed for ASA occlusion by compression of disc herniation.
